# Structural basis of sodium–potassium exchange of a human telomeric DNA quadruplex without topological conversion

**DOI:** 10.1093/nar/gku083

**Published:** 2014-01-28

**Authors:** Zi-Fu Wang, Ming-Hao Li, Shang-Te Danny Hsu, Ta-Chau Chang

**Affiliations:** ^1^Institute of Atomic and Molecular Sciences, Academia Sinica, Taipei 106, Taiwan, Republic of China, ^2^Department of Chemistry, National Taiwan University, Taipei 106, Taiwan, Republic of China, ^3^Institute of Biophotonics, National Yang-Ming University, Taipei 112, Taiwan, Republic of China, ^4^Institute of Biological Chemistry, Academia Sinica, Taipei 115, Taiwan, Republic of China and ^5^Department of Biochemical Sciences, National Taiwan University, Taipei 106, Taiwan, Republic of China

## Abstract

Understanding the mechanism of Na^+^/K^+^-dependent spectral conversion of human telomeric G-quadruplex (G4) sequences has been limited not only because of the structural polymorphism but also the lack of sufficient structural information at different stages along the conversion process for one given oligonucleotide. In this work, we have determined the topology of the Na^+^ form of Tel23 G4, which is the same hybrid form as the K^+^ form of Tel23 G4 despite the distinct spectral patterns in their respective nuclear magnetic resonance (NMR) and circular dichroism spectra. The spectral difference, particularly the well-resolved imino proton NMR signals, allows us to monitor the structural conversion from Na^+^ form to K^+^ form during Na^+^/K^+^ exchange. Time-resolved NMR experiments of hydrogen–deuterium exchange and hybridization clearly exclude involvement of the global unfolding for the fast Na^+^/K^+^ spectral conversion. In addition, the K^+^ titration monitored by NMR reveals that the Na^+^/K^+^ exchange in Tel23 G4 is a two-step process. The addition of K^+^ significantly stabilizes the unfolding kinetics of Tel23 G4. These results offer a possible explanation of rapid spectral conversion of Na^+^/K^+^ exchange and insight into the mechanism of Na^+^/K^+^ structural conversion in human telomeric G4s.

## INTRODUCTION

A G-rich single stranded DNA of telomere can form various G4 structures through Hoogsteen hydrogen bonds in the presence of monovalent cations such as Na^+^ or K^+^ (1,2). Such G4 structures have been shown to potentially exist in human chromosome end (3–7), exhibiting the ability to inhibit telomerase activity and are therefore potential targets for anticancer drug design (8–11). However, G4 structure-based drug design remains challenging because human telomeric G-rich sequences can adopt various G4 structures and possibly interconvert between them on changes in solvent compositions (12–14). For example, a nuclear magnetic resonance (NMR) study showed that Tel22, a 22-nt human telomeric sequence, d[AG_3_(T_2_AG_3_)_3_], forms an antiparallel basket G4 structure in Na^+^ solution (15), while X-ray crystallography showed that the same sequence adopts a parallel propeller G4 structure in presence of K^+^ condition (16). In addition, NMR analysis showed that Tel23 (d[TAG_3_(T_2_AG_3_)_3_]) and Tel25 (d[TAG_3_(T_2_AG_3_)_3_TT]), which contain two additional thymine nucleotides at the 3′ end, adopted different conformations in K^+^ solution, namely (3 + 1) hybrid-I and hybrid-II forms, respectively (17,18). Similar G4 structures were also observed for other truncated sequences of human telomere (19–21). Unlike other G4 structures that contain three G-tetrad layers, NF3 (d[G_3_(T_2_AG_3_)_3_T]) forms a basket antiparallel G4 structure with only two G-quartet layers in K^+^ solution (22). Recently, a newly resolved structure of human telomere G4, Tel27 (d[TTAG_3_(T_2_AG_3_)_3_TTA]), has been found to adopt a (2 + 2) topology with two lateral and one double reversal loops in Na^+^ solution (23). These findings exemplify the fact that slight sequence variations in human telomeres can result in diverse G4s with different folding topologies. Such structural diversities in human telomere G4s observed *in vitro* may possibly be present *in vivo* (24,25).

Metal ions can stabilize G4 structures by coordinating the O6 atom in the channel of G-quartet (1,2,26). Different cations can result in different spectral properties corresponding to different topologies for the same G4-forming sequences (27–30). They can also trigger conformational switching as a result of ion exchange (31). In particular, K^+^ and Na^+^ are the most physiologically relevant ions in the context of G4 structures. Sen *et al.* first demonstrated that Na^+^/K^+^ exchange can induce conformational switching (32). The ion-dependent conformational switching may be functionally important in regulating specific biological process. Understanding the underlying principle of the structural conversion of G4 induced by Na^+^/K^+^ exchange will have important implications not only in biomedical applications (33) but also for designs of G4-based nanomaterials (26,34).

Thermodynamic analysis of Na^+^/K^+^ and 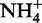/K^+^ conversion of a dimeric G4 forming sequence, d[G_3_T_4_G_3_]_2_ and d[G_3_T_4_G_4_]_2_, showed that K^+^ exerts more stabilizing effect on G4 than does Na^+^, but no structural conversion is involved during exchange (35–37). A conclusion was drawn based on their findings that the preferential K^+^ uptake over Na^+^ is driven by hydration energy (35). Concerning Na^+^/K^+^ exchange–induced structural conversions in human telomeric G4 structures, recent NMR and circular dichroism (CD) studies reported a rapid spectral conversion from the Na^+^-bound state to K^+^-bound state (hereafter referred to as the Na^+^ form and the K^+^ form) for Tel22 on K^+^ titration (19). Several explanations for the observed spectral changes were proposed (19,38–42). Yang *et al.* reported the G4 solution structure of a variant of Tel22, Tel26-M (d[AAAGGG(TTAGGG)_3_AA]), in K^+^ solution and proposed a mechanism for the Na^+^/K^+^ spectral conversion resulting from structural conversion via reorientation of a strand segment (19). Gray *et al.* used time-dependent CD spectra for monitoring kinetics of the spectral conversion of Tel22 and proposed a mechanism involving triplex intermediates for Na^+^/K^+^ conversion (42). However, the structures of Tel26-M G4 in the Na^+^ form and Tel22 G4 in the K^+^ form have not been determined.

We have used ensemble and single molecule experiments to investigate to Na^+^/K^+^ spectral conversion (39,43). We first used a G4 ligand, 3,6-Bis(1-methyl-4-vinylpyridinium) carbazole diiodide, to monitor the Na^+^/K^+^ spectral conversion in ensemble experiments using CD spectroscopy (39,43). We observed CD spectral changes of human telomeric G4, Tel24 (d[TTAG_3_(T_2_AG_3_)_3_]), during Na^+^/K^+^ exchange, but not the induced CD spectral change of ligand binding, implying that global unfolding is not involved in the Na^+^/K^+^ exchange because the G4 ligand remains bound to the G4 (39). In addition, single molecule (tethered particle motion) results showed that the Tel22 G4 underwent Na^+^/K^+^ conversion without populating distinct unfolding state (43). Recently, Vorlickova *et al.* demonstrated that for telomere G4s that have different flanking groups at the 5′ or 3′ ends can adopt the same topology, while their respective CD spectra, particularly the band at 265 nm, are different (40). These findings highlight the potential pitfall of using CD spectroscopy alone to investigate topological differences in G4s. It is therefore crucial to define the topologies of the same G4 sequence in the presence of Na^+^ and K^+^ as reference points by other means, such as NMR spectroscopy, before commencing detailed structural investigations into the process of Na^+^/K^+^ exchange–induced spectral conversion.

Although a handful of reports on the structures of different human telomeric G4s have been documented, none of the human telomeric G4s have been structurally characterized in both the Na^+^ and K^+^ forms as reference points for the structural analysis of Na^+^/K^+^ conversion. Here we use the widely studied G4 sequence of Tel23, which forms the hybrid-I type G4 structure in K^+^ solution (17), as a model system to investigate the spectral conversion induced by Na^+^/K^+^ exchange. Using NMR spectroscopy, we determine the topology of Tel23 in the Na^+^ form, which is the same as the previously reported K^+^ form topology. We further characterize the hydrogen*–*deuterium exchange (HDX) and complementary strain hybridization kinetics of Tel23 in the context of Na^+^/K^+^ spectral conversion to verify whether global unfolding of the G4 structure is associated with the Na^+^/K^+^ spectral conversion. In addition, a stepwise mechanism is proposed for Na^+^/K^+^ conversion of the Tel23 G4 based on the K^+^ titration results.

## MATERIALS AND METHODS

### DNA preparation

All unlabeled oligonucleotides were purchased from Bio Basic (Ontario, Canada). The DNA concentrations were determined by the absorption at 260 nm peaks using a UV-Vis absorption spectrometer (Nano-viewer, GE Healthcare, USA). The oligonucleotides were dissolved in 10 mM Tris–HCl (pH 7.5) and 150 mM NaCl and/or KCl, followed by heat-denaturation at 95°C for 5 min and slowly annealed to room temperature (1 min/°C). The annealed oligonucleotides were stored at 4°C at least 1 day before experiments. The site-specific ^15^N-labeled oligonucleotides (44) were synthesized using a solid-phase oligonucleotide synthesizer (Dr Oligo, USA). The ^15^N-labeled dG-phosphoramidite (Cambridge Isotope Laboratories, USA) was mixed with unlabeled dG-phosphoramidite (Sigma, USA) to make up a stock solution of 1.0 M dG-phosphoramidite in acetonitrile enriched with 6% ^15^N-material. It was then used together with unlabeled dA and dT-phosphoramidite to synthesize site-specific ^15^N-labeled oligonucleotides using solid-phase *β*-cyanoethyl phosphoramidite chemistry of which 6% of the material was ^15^N-labeled.

### CD spectroscopy

The CD experiments were conducted using a spectropolarimeter (J-815, Jasco, Japan) with a bandwidth of 2 nm at a scan speed of 50 nm/min and a step resolution of 0.2 nm over the spectral range of 210–350 nm. The strand DNA sample concentration was 5 µM in specific buffer conditions. The thermal melting curves were recorded by a peltier thermal coupler chamber (PFD-425S/15, Jasco, Japan), and the molar ellipticity was monitored at 295 nm between 25 and 95°C with a temperature ramping rate of 1°C/min rate. The observed signals were baseline subtracted, and the first derivative zero points were defined as the melting temperature.

### NMR spectroscopy

All NMR experiments were performed on a Bruker AVIII 500 MHz and AVIII 800 MHz spectrometers (Bruker, USA), equipped with a Prodigy and a cryogenic probe, respectively. One-dimensional imino proton NMR spectra in the chemical shift range of 10–13 ppm were recorded using a WATERGATE pulsed sequence or a jump-and-return scheme (45). The 1D ^15^N-^1^H SOFAST-HMQC spectra were used to unambiguously determine the assignments of individual imino proton resonances from the site-specifically ^15^N-labeled NMR samples (46), which of each sample contains 6% ^15^N-labeled guanine base at one of the 11 G-tetrad-forming guanine residues as described above (44,47). All NMR samples were dissolved containing 10% D_2_O with 150 mM NaCl and/or KCl. The strand concentrations of the NMR samples were typically 0.5–1 mM in specific salt conditions with an internal reference of 0.1 mM 4,4-dimethyl-4-silapentane-1-sulfonic acid. The nuclear overhause effect spectroscopy (NOESY) spectra of exchange and nonexchange inter-proton were assigned using SPRKY software (http://www.cgl.ucsf.edu/home/sparky/). Exchange inter-proton distances were calculated from the initial slopes of NOE buildup curves for NOESY spectra recorded at mixing times of 50, 100, 150 and 200 ms in the Na^+^, K^+^ or Na^+^/K^+^ forms. The Na^+^/K^+^ form was generated from the Na^+^ form after addition of equal amount K^+^ ion to identify NOE crosspeaks due to spin diffusion. The NOESY spectra of nonexchange inter-proton distances were recorded in D_2_O at 200 ms. The inter-proton distances were derived from the NOE intensities with respect to those of thymine H6-CH3, which has a fixed distance of 2.99 Å and was used as the reference distance (15,18,19).

### Equilibrium titrations

The fractional populations of the Na^+^ or K^+^ forms were determined by measuring changes in imino proton NMR as a function of K^+^ ion concentration. The titration data were fitted by one site binding model using nonlinear least squares module of the program Origin 7.5 (OriginLab Corp, USA) to optimize values. The fractions of [a] and [c] correspond to the normalized intensity of imino proton in the Na^+^ and the K^+^ forms, and plotted as a function of K^+^ ion concentration to calculate the equilibrium constant of K_1_ and K_2_ (Figure 8 and Supplementary Figure S5A). The following are the fitting equations:

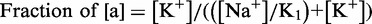


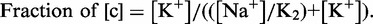


### NMR kinetic measurements

The Tel23 G4 oligonucleotides samples for the HDX exchange experiments were prepared in 150 mM Na^+^, 10 mM Tris–HCl (pH 7.5), followed by an anneal procedure as described above, and then lyophilized. The lyophilized oligonucleotides were resuspended in 99% D_2_O with (pretreat or simultaneous) or without 150 mM K^+^ to reach the DNA concentration of 100–200 µM, immediately before NMR measurements. For the hybridization experiments, the oligonucleotides of Tel23 (d[TAGGG(TTAGGG)_3_]) and C23 (d[(CCCTAA)_3_CCCAT]) were prepared in 150 mM Na^+^, with a strand concentration of 200 µM. The experiment was performed by mixing C23 sample with or without K^+^ 150 mM into equal amount of Tel23 sample and NMR signal of imino proton region was recorded immediately.

## RESULTS

### Spectral conversion of Tel23 G4 induced by Na^+^/K^+^ exchange

**Figure 1.**
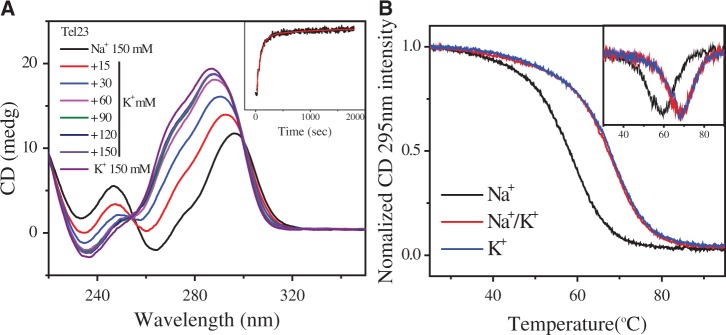
(**A**) CD spectra of 10 µM Tel23 G4 in Na^+^ solution on K^+^ titration at 25°C. The insert shows the real-time CD signal at 265 nm after adding 150 mM K^+^ into 150 mM Na^+^ solution and the curve was fitted by bi-exponential equation. (**B**) CD thermal melting curves of Tel23 G4 in the Na^+^ (black), K^+^ (blue) and Na^+^/K^+^ (red) forms monitored at 295 nm. The inset shows the first derivative of the melting curves.

CD spectroscopy is a popular tool for studying G4 structures (26,48,49). In general, parallel G4 structures, such as the propeller form, exhibit a positive band at 265 nm and a negative band at 240 nm; antiparallel G4 structures, such as the basket and the chair forms, exhibit two positive bands at 295 and 240 nm, together with a negative band at 265 nm. These spectral features are mainly attributed to different modes of guanine stacking in different G4 topologies (50,51). Figure 1A shows the CD spectra of Tel23 in 150 mM Na^+^ solution on K^+^ titration. Each spectrum was recorded right after the K^+^ titration. Our results clearly show a rapid spectral conversion from the Na^+^ form to the K^+^ form. In addition, an isosbestic point at 255 nm exists on K^+^ titration into the Na^+^ form, implying a two-state process for the Na^+^/K^+^ spectral conversion of Tel23 G4. The inset shows the real time CD signal at 265 nm after adding 150 mM K^+^ into 150 mM Na^+^ solution. The biphasic transition curve can be fitted by two arising time constants of ∼80 s with A_1_ = 89% and ∼930 s with A_2_ = 11% (R^2 ^= 0.98). Figure 1B shows the CD melting curves of the Na^+^ and K^+^ forms. The melting temperature (Tm) was determined by the first derivative of the sigmoidal CD melting at 295 nm. The Tm of the K^+^ form Tel23 is 68.9°C, which is higher than that of the Na^+^ form (59.3°C). Addition of K^+^ into the Na^+^ form leads to a large increase of Tm (68.9°C), which is consistent with the previous results (19,39,42). The fact that Tel23 G4 preferentially uptakes K^+^ over Na^+^ ion is in line with the results of other human telomere G4 systems (19,38–42).

### Topology of Tel23 G4 in the Na^+^ form

**Figure 2.**
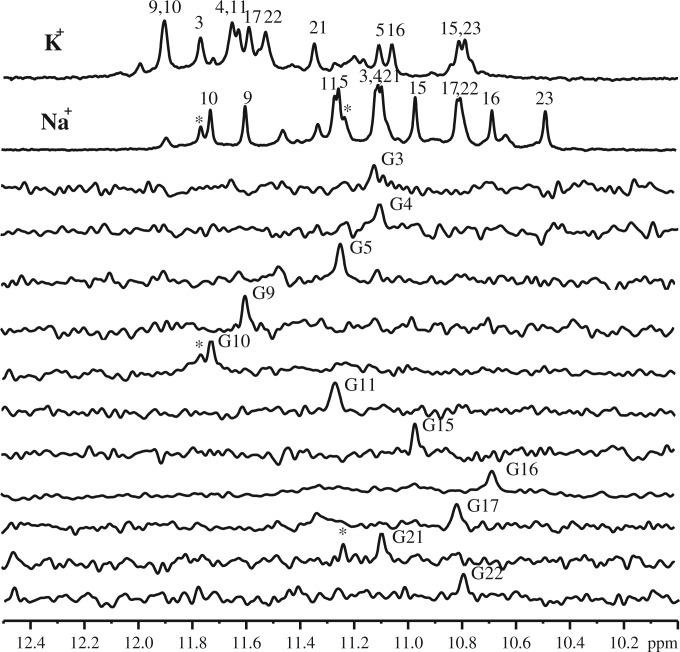
Site-specific assignments of imino proton resonances of Tel23 G4 in the Na^+^ form. The imino proton NMR spectra of Tel23 G4 in the K^+^ and Na^+^ forms are shown in the top first and second rows of stacked spectra, respectively. The residue-specific assignments are labeled on top of individual peaks. The 1D ^15^N-^1^H SOFAST-HMQC spectra of 6% ^15^N enriched Tel23 G4 samples are shown below with the assignments and site-specific labeled that corresponds to labeling sites. (The star signals are minor conformation peaks). All NMR spectra were recorded at 25°C. The assignments of the K^+^ form are taken from (17).

A previous NMR study showed that Tel23 adopts a hybrid-I–type G4 structure in the K^+^ form (17), but the topology of the Na^+^ form of Tel23 is hitherto undetermined. Here, we used multidimensional NMR spectroscopy to determine the topology of Tel23 in Na^+^ solution and compared the imino proton NMR spectra of Tel23 G4 in the Na^+^ and K^+^ forms. Both of them exhibit distinct imino proton resonances between 10.5 and 12 ppm (Figure 2) that can be used to determine the major G4 structures for both forms. In comparison, the Na^+^ form of Tel22, which lacks a thymine at 5′ end compared with Tel23, adopts an antiparallel basket structure (15). Despite the small difference in their sequences, the imino proton NMR spectrum of Tel22 in the Na^+^ form is different from that of Tel23 (Supplementary Figure S1). Therefore, it is likely that the topologies of the two G4s are different. To unambiguously establish the topology of Tel23 in the Na^+^ form, we embarked on the elucidation of the Na^+^ form of Tel23 using NMR spectroscopy.

**Figure 3.**
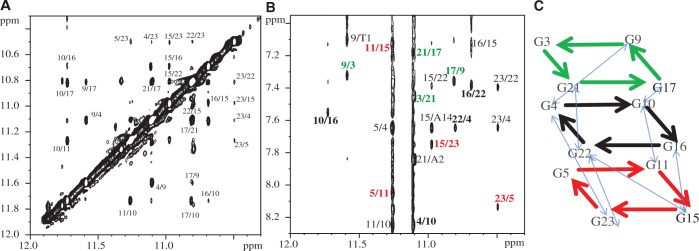
Determination of Tel23 G4 topology in Na^+^ solution. Guanine imino (H1)–imino (H1) proton (**A**) and imino (H1)–aromatic (H8) proton (**B**) regions of NOESY spectrum of Tel23 G4 in the Na^+^ form. The NOESY spectrum was recorded at 18.6 T and 25°C with a mixing time of 200 ms. The crosspeaks that correspond to the NOE connectives within the three G-tetrads (colored green, red and black) are boxed and labeled with the residue number of imino proton and that of aromatic proton. (**C**) Observed guanine NOE connectives of Tel23 G4. The intra-tetrads NOEs are shown in thick lines and colored as in (B). Guanine imino (H1)–aromatic (H8) NOE connectives observed are G3→G21→G17→G9 (green), G4→G10→G16→G22 (black) and G5→G11→G15→G23 (red). The inter-tetrad NOEs are shown in blue lines.

**Figure 4.**
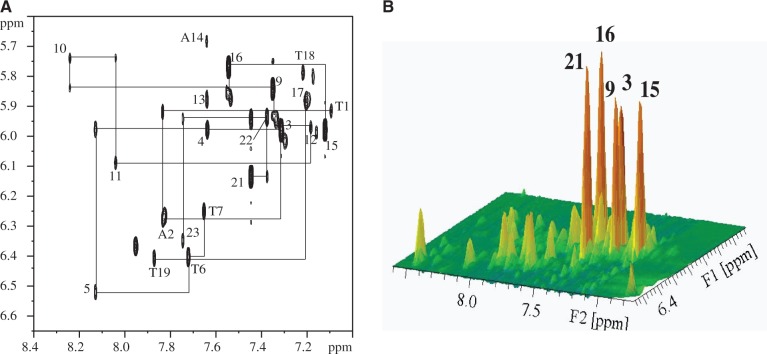
Glycosidic configurations of tetrad-forming guanines. (**A**) Rectangular H8-H1’ patterns for 5′-syn-anti-3′ steps are highlighted by black lines with specific guanine assignment. (**B**) Stacked plot of the same expanded NOESY spectrum of Tel23 G4 to highlight the five strong intra-residue H8-H1′ crosspeaks, which correspond to the ‘syn’ glycosidic bonds of Gx, Gy, Gz … , (xyz are specific guanine assignment), while the others that exhibit weak crosspeaks correspond to ‘anti’ glycosidic bonds.

We applied the sequence-specific assignments of the imino proton NMR spectra to determine the Na^+^ form of Tel23 topology. Eleven Tel23 samples were synthesized, each of which is site-specifically labeled with ^15^N-enriched guanine. One-dimensional ^15^N-edited SOFAST-HMQC spectrum was recorded for each of these samples to obtain site-specific assignments of the imino proton resonances (Figure 2) and those of the aromatic H8 protons (Supplementary Figure S2) (46). It should be noted that there are some additional imino signals observed in Figure 2, implying the possible existence of other minor conformations. Because these additional signals are weak and cannot be identified in the isotope-labeling experiment, we therefore only consider the major conformation of Tel23 in this work. NOESY was recorded to define the topology of Tel23 G4 in the Na^+^ form by establishing the through space connectives between individual imino and aromatic protons (Figure 3A). Based on the NOEs between the H1 imino protons and H8 protons, we could confirm the topology (Figure 3B). The Na^+^ form of Tel23 G4 consists of three G-tetrads: the top G-tetrad is anticlockwise (G3→G21→G17→G9), while the middle (G4→G10→G16→G22) and bottom (G5→G11→G15→G23) G-tetrads are clockwise (Figure 3C). As such, the first loop (T6-T7-A8) adopts a double-chain-reversal configuration, while the second (T12-T13-A14) and third loops (T18-T19-A20) adopt lateral configurations. These results show that the Na^+^ form of Tel23 G4 adopts a hybrid-I type topology, with one G-strand running in the opposite direction with respect to the other three. In addition, we determine the glycosidic conformations to be asymmetrically oriented by the H1’-H8 NOE patterns (Figure 4), where the four glycosidic bonds in the top G-tetrad are syn-syn-anti-syn, while those of the other two G-tetrads are anti-anti-syn-anti. The syn guanine bases are G3, G9, G21, G15 and G16, which exhibit strong H1’-H8 NOEs (Figure 4B) and are the same as the Tel23 K^+^ form (17). Because the topology of Tel23 is similar in both forms, why do their respective CD spectra show such differences? We therefore quantitatively analyze NOE signals of both forms (Supplementary Tables S1 and S2), the overall G-tetrads of the Na^+^ form (average distances in Å are 5.1 ± 0.2 for H8-H1, and 4.9 ± 0.6 for H1-H1) are more loosely stacked than those of the K^+^ form [average distances in Å are 4.6 ± 0.4 for H8-H1 and 4.3 ± 0.3 for H1-H1; the difference is statistically significant (*P* < 0.05)]. In addition, some loop conformations show greater distances in Na^+^ form than K^+^ form (Supplementary Table S2). However, the glycosidic conformations of H1’-H8 exhibit no obvious differences between the two forms. Most importantly, after Na^+^/K^+^ ion exchange, the H8-H1 and H1-H1 distances within the G-tetrads become shorter (average distance in Å are 4.3 ± 0.4 for H8-H1 and 4.0 ± 0.3 for H1-H1) and are similar to those in the K^+^ form. These results suggest that the Na^+^ and K^+^ forms contain different distances in base stacking, which could possibly give rise to spectral differences although they share the same topology. We therefore concluded that the major topology of the Tel23 Na^+^ form is the same hybrid-I type G4 type as the major K^+^ form determined previously (17).

### HDX and hybridization NMR studies

**Figure 5.**
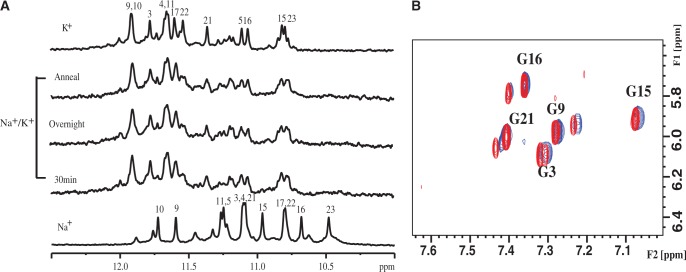
Na^+^/K^+^ conversion in Tel23 G4 monitored by NMR spectroscopy. (**A**) The imino proton spectra of Tel23 G4 in the Na^+^ form, 30 min after the addition of equal amount of K^+^, overnight incubation, after annealing process and in the K^+^ form are shown in ascending order. (**B**) Overlay of the H8-H1’ region of the NOESY spectra of Tel23 G4 in the K^+^ form (red) and that recorded 30 min after the addition of equal amount of 150 mM K^+^ into the Na^+^ form (blue). The results indicated that the spectral change induced by Na^+^/K^+^ exchange is completed within 30 min after the additions of K^+^ ion to the Na^+^ form of Tel23 G4.

Given that the topology of Tel23 G4 of the Na^+^ form is the same as the K^+^ form, the next question is whether unfolding of the G4 structure is involved in the Na^+^/K^+^ spectral conversion. We compared the imino proton NMR spectra of Tel23 G4 after the addition of K^+^ to the Na^+^ form. The imino proton resonances converted from the Na^+^ form to the K^+^ form within 30 min (Figure 5A, B). To determine the timescale of the Na^+^/K^+^ exchange process, we therefore recorded the imino proton signals of the first NMR spectrum immediately after the addition of K^+^ ion. The first NMR spectrum of the Na^+^/K^+^ form of Tel23 was recorded after 85 s and this spectral pattern is identical to the one recorded 30 min after the addition of K^+^ (Supplementary Figure S3), suggesting that the Na^+^/K^+^ exchange is a rapid process, which takes place within tens of seconds (42).

**Figure 6.**
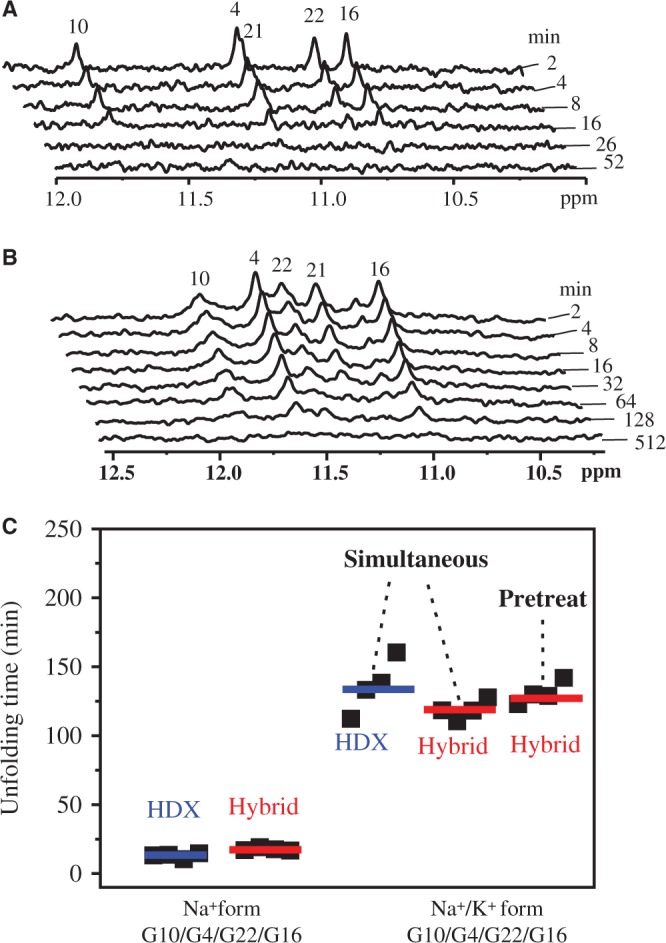
NMR HDX kinetics of Tel23 G4 in the Na^+^ and the simultaneous Na^+^/K^+^ forms. (**A**) The Na^+^ form sample was lyophilized and then dissolved in 99% D_2_O immediately before NMR measurement. (**B**) The same sample preparation procedure as in (A), but with equal amount of 150 mM K^+^ added together with D_2_O, named the simultaneous Na^+^/K^+^ form. (**C**) The unfolding time constant of Tel23 G4 in the Na^+^, the simultaneous Na^+^/K^+^ (200 μM of the Na^+^ form sample was prepared in 150 mM K^+^ added together with 200 μM complementary strand of Tel23) and pretreat Na^+^/K^+^ forms (the Na^+^ form sample was added 150 mM K^+^, and then equal amount of complementary strand of Tel23 was added), derived from the decays of HDX and hybridization experiments (Supplementary Figure S4).

We then asked the question of whether there is an unfolded state, partially or totally, during the kinetic process of Na^+^/K^+^ exchange. Real-time NMR HDX experiments have been used to study the unfolding kinetics of G4s at a resolution of individual guanines (52,53). Each HDX rate of the imino protons reveals solvent exposure of the dynamics of individual hydrogen bond within the G-tetrads and provides unfolding kinetics. The slower the exchange rate, the more protected the hydrogen bond donor, i.e. imino group within G-tetrads. In general, the imino protons in the top and bottom layers of G4 exchange much faster than those in the middle layer of a G4 structure, which is sequestered from bulk deuterated solvent by the top and bottom layers. Immediately after dissolving the Na^+^ form of Tel23 G4 in D_2_O buffer, only four resonances remained in the NMR spectrum, which correspond to the four imino protons in the central G-tetrads (Figure 6 and Supplementary Figure S4). The HDX time constants of the four central imino protons in the Na^+^ form are equally short (14 ± 5 min; Figure 6C). In contrast, the HDX time constants of the four imino protons in the central G-tetrad of the simultaneous Na^+^/K^+^ form of Tel23 G4, which is prepared by simultaneous addition of K^+^ ion and D_2_O buffer into Na^+^ form, are much longer (136 ± 28 min; Figure 6C and Supplementary Figure S4), indicating that addition of K^+^ ion can further stabilize Tel23 G4.

To further investigate the unfolding kinetics of Tel23, we used a complementary sequence C23, d[(CCCTAA)_3_CCCTA], to carry out real-time hybridization experiments by NMR (Supplementary Figure S4). During hybridization, the formation of a DNA duplex can be monitored by the appearance of down-field shifted resonances at 13–14 ppm, which correspond to Watson–Crick base-paired imino protons. This is accompanied by the loss of G4 imino proton signals (54,55). Assuming that the complementary C23 strand functions as a bait without perturbing the unfolding kinetics of Tel23 G4, one can obtain the global unfolding kinetics of the G4 structure by monitoring the decay of individual G4 imino proton resonances. The hybridization results show that the unfolding time constants, 17 ± 5 min for the Na^+^ form and 127 ± 16 min for the simultaneous Na^+^/K^+^ form (simultaneous addition of K^+^ and complementary strand into the Na^+^ form), are similar to those of HDX rate determining time constants, 14 ± 5 min for the Na^+^ form and 136 ± 28 min for the simultaneous Na^+^/K^+^ form (simultaneous addition of K^+^ and D_2_O into the Na^+^ form), respectively (Figure 6C and Supplementary Figure S4). Moreover, we compared the simultaneous Na^+^/K^+^ form with the pretreated Na^+^/K^+^ form (130 ± 13 min), which is formed by the addition of K^+^ ion before the addition of complementary strand into Na^+^ form. The results show that the time constants in the two hybridization experiments are nearly identical (Figure 6C). We therefore conclude that the rapid spectral conversion induced by Na^+^/K^+^ exchange does not involve the global unfolding of Tel23.

### Spectral conversion of Tel23 G4 Na^+^ form on K^+^ titration

**Figure 7.**
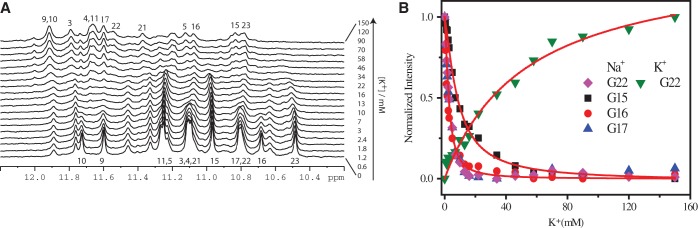
(**A**) Imino proton NMR spectra of Tel23 G4 in 150 mM Na^+^ on K^+^ titration. (**B**) The intensities of G15, G16 and G17 imino protons in the Na^+^ form and G22 in both the Na^+^ and K^+^ forms as a function of K^+^ concentration.

Because both CD and NMR spectra of Tel23 G4 show significant changes on the addition of K^+^, we then asked the question of how the replacement of Na^+^ by K^+^ in Tel23 G4 induces the observed spectral conversion. Several studies have suggested a step-by-step mechanism for the replacements of Na^+^/K^+^ and 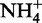/K^+^ during ion exchange of dimeric G4s (35,37). To monitor the Na^+^/K^+^ exchange at a residue-specific level, an NMR-based K^+^ titration experiment was undertaken for Tel23 G4 to monitor the ion exchange process from the Na^+^ form to the Na^+^/K^+^ form (Figure 7A). The K^+^ titration data show that the Na^+^ form signals decrease more rapidly than the increase of the K^+^ form signals, suggesting the presence of an intermediate, which is a mixed di-cation form (37). The ion exchange from pure Na^+^ through a mixed Na^+^-K^+^ form to pure K^+^ forms can be expressed by the two equilibrium constants (37),

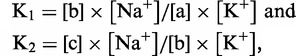

**Figure 8.**
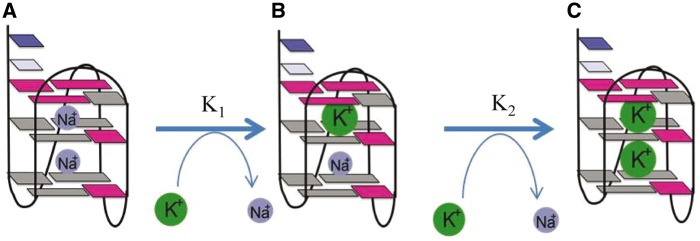
Proposed mechanism of Na^+^/K^+^ exchange process of Tel23 G4s. The pure Na^+^ form as (**A**), the mixed Na^+^–K^+^ form as (**B**) and the pure K^+^ form as (**C**).

 Where a, b and c correspond to the three di-cation forms defined in Figure 8 and the sum of a, b and c equals to the total amount of folded structure. By fitting the titration curves of individual imino protons (Figure 7B), we obtained the equilibrium constants from the disappearance of the Na^+^ form signals (K_1_) and the appearance of the K^+^ form signals (K_2_) (Supplementary Table S1). The equilibrium constant K_1_ = 59.7 is larger than K_2_ = 2.7 for G22, indicating that the first K^+^ ion binding is more favorable than the second one, which is consistent with 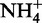/K^+^ exchange results (37). Using K^+^ ion concentration as the log function, Supplementary Figure S5A shows the curve of the intermediate (b), which can be obtained by subtracting (a) and (c) from the total amount of folded structure. Furthermore, according to the disappearance of the Na^+^ form, most of the imino proton resonances in bottom G-tetrad are less affected than others at low K^+^ concentration, especially G5 and G15 (Supplementary Table S3). The K_1_ of G15 is much smaller than other imino protons on top and central G-tetrads (Figure 7B, Supplementary Figure S5B). It is suggested that Na^+^/K^+^ exchange is a step-by-step process that involves an intermediate state with two different sites, where the upper site between the top and central G-tetrad exchange more favorably than the lower site, i.e. the exchange may not occur simultaneously (37). In other words, the Na^+^/K^+^ exchange process in Tel23 is likely a stepwise process that does not involve global unfolding.

## DISCUSSION

In this work, we have determined the major topology of the Na^+^ form of Tel23 G4 to be the hybrid-I type topology, which is the same as that of the K^+^ form. With a single-nucleotide difference at the 5′ end, the Na^+^ form of Tel22 G4 adopts the basket type topology with the same syn-syn-anti-anti arrangements of the glycosidic angles together with successive lateral-diagonal-lateral connecting loops. Patel *et al.* showed that the three-repeat human telomeric sequence d(GGGTTAGGGTTAGGGT) can associate with the single-repeat human telomeric sequence d(TAGGGT) to form a (3 + 1) asymmetric dimeric G-quadruplex in Na^+^ solution with a similar topological arrangement as what we reported here for the Tel23 in the Na^+^ form (56). The Na^+^ form of the four-repeat ‘Tetrahymena’ telomeric sequence d[(TTGGGG)_4_] adopts a (3 + 1) hybrid-II topology (57). Recently, Tel27 (d[TTAG_3_(T_2_AG_3_)_3_TTA]) is found to adopt a (2 + 2) topology with two lateral and one double reversal loops (23). These results illustrate the structural polymorphism of G4s, which is sensitive to sequence compositions as well as experimental conditions.

Although the Na^+^ form of Tel23 could adopt same topology as K^+^ form, why do their respective spectra show large differences? Schultze *et al.* (36) found that dimeric antiparallel G4 of ‘Oxytricha’ telomere oligonucleotide d(G_4_T_4_G_4_)_2_ showed difference in the T4 loop conformation between the Na^+^ and K^+^ forms even though they adopted same topology. In addition, Vorlickova *et al.* (40,44) systematically studied CD spetra of different G4 oligonucleotides, where they suggested CD spectra are sensitive to base stacking. The changes in G-tetrads stacking or the addition of an adenine base at the 5′ or 3′ end, in addition to differences in glycosidic conformations and loop conformations, could result in significant CD spectral changes (36,40,44). The H8-H1 and H1-H1 distances derived from the observed NOE crosspeaks show small but statistically significant differences in the guanine stacking distance and loop conformations between the Na^+^ and K^+^ forms, which may explain the observed differences in the observed CD spectra. It is also likely that the minor population that is observed in imino proton NMR spectra of Tel23 of which the topology is uncharacterized, can contribute to the ensemble CD signal. The imino proton NMR signals provide the details of individual G-bases of the G4 during the ion exchange process, while CD provides information on the ensemble signal of the sample. These minor conformations may be different in Na^+^ and K^+^ forms, which could contribute to different ensemble CD signals.

Na^+^/K^+^ exchange process of human telomeric G4 has been investigated by many research groups. Yang *et al.* have characterized a telomeric sequence, Tel26M with flanking bases at the 5′ and 3′ ends, which adopts a hybrid-I type topology in the K^+^ form (19). An assumption was made, based on the CD data, that the topology of the Na^+^ form of Tel26M is the same as that of Tel22 G4, which adopts mainly a basket-type G4 topology in the Na^+^ form. Because of the topological differences, they proposed that the Na^+^/K^+^ conversion may involve structural dissociation and rearrangement. Gray *et al.* have monitored real-time CD signals at 291 nm and 265 nm of Tel22 in 30 mM Na^+^ solution after the addition of 50 mM K^+^ (42). They found a rapid change in the observed CD signal within the first 5 s of the instrumental dead time followed by two kinetic phases with their respective time constants of 40–50 s and 600–800 s at 25°C. Accordingly, they proposed a kinetic model for the Na^+^/K^+^ exchange–induced structural conversion from the Na^+^ basket form to the K^+^ hybrid form through a Na→I_1_→I_2_→K pathway. They suggested that a fast ion exchange within the 5-s experimental dead time, followed by the formation of a partially unfolded triplex structure (I_1_) with a relaxation time of ∼50 s, and a triplex folding intermediate (I_2_) to the hybrid conformation with a slower relaxation time of ∼800 s.

The biphasic time constants of Na^+^/K^+^ exchange of Tel23 G4 are ∼80 and 930 s, respectively, which are in the same orders as the two time constants for the Na^+^/K^+^ exchange of Tel22 G4 with 50 and 800 s (42). Our NMR data supported that Tel23 adopts the same hybrid-I type topology in both Na^+^ and K^+^ forms despite the large spectral differences in their respective CD and NMR spectra. However, the model proposed by Gray *et al.* was based on the assumption that the two end states, namely, the initial Na^+^ form and the final K^+^ form, are different G4 topologies. The basket Na^+^ form of Tel22 G4 was determined by NMR (15), while the hybrid-I K^+^ form of Tel22 G4 was inferred from the CD spectrum (19).

NMR HDX experiments are widely used to identify resonances corresponding to imino protons at the central G-tetrad. Phan *et al.* and Hsu *et al.* previously used this method to explore the unfolding kinetic of G4 structure (52,53). By evaluating the HDX rates of imino protons, they obtained kinetic information from different G4s at the level of individual guanines. Here, we used NMR HDX to study the Na^+^/K^+^ spectral conversion and conducted experiments by adding K^+^ ion and D_2_O solvent simultaneously. Our results show that the average unfolding time constant of central G tetrads of Tel23 G4 in the Na^+^ form is only about one-tenth of that in the Na^+^/K^+^ form (Figure 6), implying that the unfolding time of the Na^+^/K^+^ form is much longer than the original Na^+^ form and that binding of K^+^ can rapidly stabilize Tel23 G4. If the Na^+^/K^+^ exchange process involved the formation of globally unfolded species, the imino proton signal of central G-tetrad should disappear as soon as K^+^ ion and D_2_O are added to the sample. In general, the HDX exchange for the duplex DNA is on the timescale of milliseconds (58), and that for fully unfolding oligonucleotides is even faster. Here, the HDX for the imino protons located at the top and bottom layer of the G-tetrads of Tel23 G4 is rapid (these resonances disappeared within the experimental dead time of our NMR experiment), despite the protection from the Hoogsteen hydrogen bonds. However, the HDX for the imino protons at central G-tetrad is much slower (14 ± 5 min; Figure 6C), implying that the central G-tetrad is protected by the top and bottom two G-tetrads. This HDX time therefore sets a lower limit for the rate of global unfolding. The results provide strong evidence to suggest that the fast spectral conversion of Tel23 G4 during Na^+^/K^+^ exchange (∼80 s) does not involve global unfolding. In addition, if the Na^+^/K^+^ exchange of Tel23 G4 involved fast global unfolding, the HDX time for Na^+^/K^+^ form would be either equal or faster than the HDX time in Na^+^ form. However, the HDX time for Na^+^/K^+^ form (136 ± 28 min; Figure 6C) is ∼10-fold longer than the HDX time for Na^+^ form (14 ± 5 min; Figure 6C). Similarly, if a triplex intermediate existed during Na^+^/K^+^ exchange of Tel23 G4, one of the imino proton signals of central G-tetrad should disappear much faster than the other. Our real-time HDX NMR results do not support such a mechanism for Na^+^/K^+^ exchange of Tel23 G4. We would emphasize that different mechanisms could be likely involved for the structural changes between different G4 topologies. Considering the structural polymorphism of telomeric G4s, it is possible that Tel22 and Tel23 have different exchange mechanisms. Nevertheless, we believe that it is necessary to determine the initial and final state before discussing the mechanism.

The use of the complementary strand DNA for the hybridization experiment by NMR is crucial to elucidate whether the unfolding is due to well-defined stepwise events or random processes. The hybridization data show no appreciable difference on the decays of the ***12*** imino proton signals, implying that the G4 unfolding is unlikely due to a sequential unfolding ***of*** events. In addition, our hybridization kinetics (127 ± 16 min; Figure 6C) provide an overall estimate of the timescale of global unfolding, which is similar to the HDX time for Na^+^/K^+^ form (136 ± 28 min; Figure 6C). We further compared the kinetics of hybridization of simultaneous addition of complementary strand and K^+^ with that of the addition of K^+^ before adding complementary strand into Na^+^ form. These two kinetic time constants are essentially the same, implying that the addition of K^+^ can kinetically stabilize the Tel23 G4 instead of induc***ing*** the global unfolding of Tel23 G4.

The Na^+^/K^+^ exchange mechanism was previously investigated by Hud *et al.* (35). Hud *et al.* monitored the change of the aromatic proton NMR signals of thymine bases in a dimeric G4 structure (G_3_T_4_G_3_)_2_ during the Na^+^/K^+^ exchange process (35). They found a two-step binding event when K^+^ ion was added. Based on the same topology of the Na^+^ form and K^+^ form being identical, they proposed a stepwise exchange process, which involves mixed Na^+^–K^+^ coexisting state. Later, Ida *et al.* observed such state in this dimeric G4 structure by ^23^Na NMR experiment (59). The studies of dynamic ion exchange in G4s were reported by Plavec *et al.* (37,60,61). They directly observed the 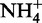 ion bound in G-tetrads, and found that the increase of K^+^ ion first leads to replacement of ^15^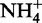 ion at the upper site of d(G_3_T_4_G_4_)_2_, followed by a full conversion into the K^+^ form. Furthermore, they determined the equilibrium constants of the two exchange processes with K_1_ to be 234 and K_2_ to be 29 and found the existence of mixed K^+^–^15^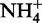 form, which are similar to the values for Tel23 as reported herein. Thus, it is possible that the mixed Na^+^–K^+^ ions are coordinated in the same G4 structure and the same process is associated with Tel23 G4.

The imino proton NMR spectra of the initial state of the Na^+^ form and the final state of the K^+^ form are well resolved (Figure 2), which enable us to directly monitor the conversion of the imino proton signals during Na^+^/K^+^ exchange. The NMR results show that the decrease of the imino proton signals of Na^+^ form is much faster than the increase of the imino proton signals of K^+^ form (Figure 7B). For example, the corresponding equilibrium constant K_1_ is large***r*** than K_2_ by 22-fold for G22 (Supplementary Table S1). Thus, the results imply that a mixed Na^+^/K^+^ coordinated G4 structure may exist and is similar to the previous results of a dimeric G4 structure (G_3_T_4_G_4_)_2_ (37). Moreover, the imino protons of the bottom G-tetrad G5, G15 and G23 remain in the Na^+^ form on addition of low K^+^ concentration (Figure 7A) and their equilibrium constant K_1_ are smaller than those of other imino protons by 2*-* to 5-fold (Supplementary Table S1). We further compared the changes of imino proton NMR intensities of the guanines in the same strand (G15-G16-G17) as a function of K^+^ ion concentration (Supplementary Figure S5B). The results indicate that G15 displays a different binding preference to the others. In other words, the top and central layers may convert to the K^+^ form concomitantly. Together, these findings lead to the proposal of the step-by-step exchange mechanism that is similar to the previous works (35,37).

In summary, our HDX and hybridization experiments demonstrated that the addition of K^+^ ion can further stabilize the G4 structure of Tel23. As a result, the fast spectral conversion of Tel23 G4 induced by Na^+^/K^+^ exchange does not involve global unfolding or triplet intermediate state.

## CONCLUSION

In this work, we have determined the topology of the Na^+^ form of Tel23 G4 to be the same hybrid form as the K^+^ form of Tel23. This represents an example of a human telomeric G4 structural study for which the topologies of both the Na^+^ and K^+^ forms of the same G4 forming DNA sequence are determined. Importantly, the topological information of the two states, the Na^+^ and K^+^ forms, serves as the reference points for our subsequent mechanistic study into the Na^+^/K^+^ spectral conversion. We have shown that the spectral conversion induced by Na^+^/K^+^ exchange possibly involves a stepwise process that starts with Na^+^/K^+^ exchange between the central and top layer. The NMR HDX and hybridization experiments show that the Na^+^/K^+^ exchange in Tel23 G4 does not involve global unfolding or triplex intermediates and that the addition of K^+^ can further stabilize Tel23 G4 quickly. It remains to be seen as to whether the same Na^+^/K^+^ conversion mechanism applies to other human telomeric G4s and possibly other DNA or RNA G4s.

## SUPPLEMENTARY DATA

Supplementary Data are available at NAR Online.

## FUNDING

Academia Sinica [AS-102-TP-A07]; National Science Council of the Republic of China [NSC-101-2113-M001-022 to T.C.C.]; Career Development Award [CDA - 00025/2010-C] from the International Human Frontier Science Program and National Science Council [100-2113-M-001-031-MY2, 101-2627-M-001-004 and 102-2113-M-001-017-MY2] and Academia Sinica, Taiwan (to S.T.D.H.). Funding for open access charge: Academia Sinica [AS-102-TP-A07].

*Conflict of interest statement*. None declared.

## Supplementary Material

Supplementary Data
