# Supplementary material for: Structural basis of sodium–potassium exchange of a human telomeric DNA quadruplex without topological conversion
Source: Nucleic Acids Res. 2014 Jan 28;42(7):4723–33. doi: 10.1093/nar/gku083 (PMC3985656; doi:10.1093/nar/gku083)
Supplement: Supplementary Data [file supp_42_7_4723__index.html]

Structural basis of sodium–potassium exchange of a human telomeric DNA quadruplex without topological conversion — Supplementary Data 

# Structural basis of sodium–potassium exchange of a human telomeric DNA quadruplex without topological conversion

## Supplementary Data

files

**Files in this Data Supplement:**

- Supplementary Data - pdf file
