# Supplementary material for: Structural basis of sodium–potassium exchange of a human telomeric DNA quadruplex without topological conversion
Source: Nucleic Acids Res. 2014 Jan 28;42(7):4723–33. doi: 10.1093/nar/gku083 (PMC3985656; doi:10.1093/nar/gku083)
Supplement: Supplementary Data [file supp_gku083_nar-03095-f-2013-File003.pdf]

Figure S1

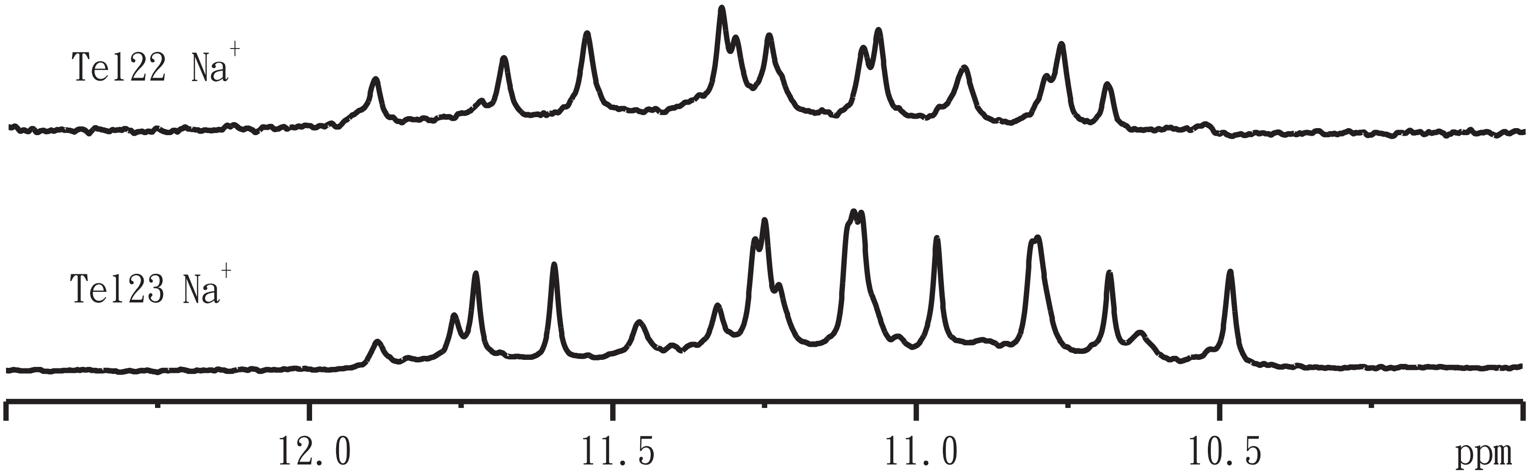

Figure S1. Imino proton NMR spectra of human telomeric G4 forming sequences with different flanking base in 150 mM Na^+ solution: Tel23 (d[TAGGG(TTAGGG)$_3$]) and Tel22 (d[AGGG(TTAGGG)$_3$]).

Figure S2

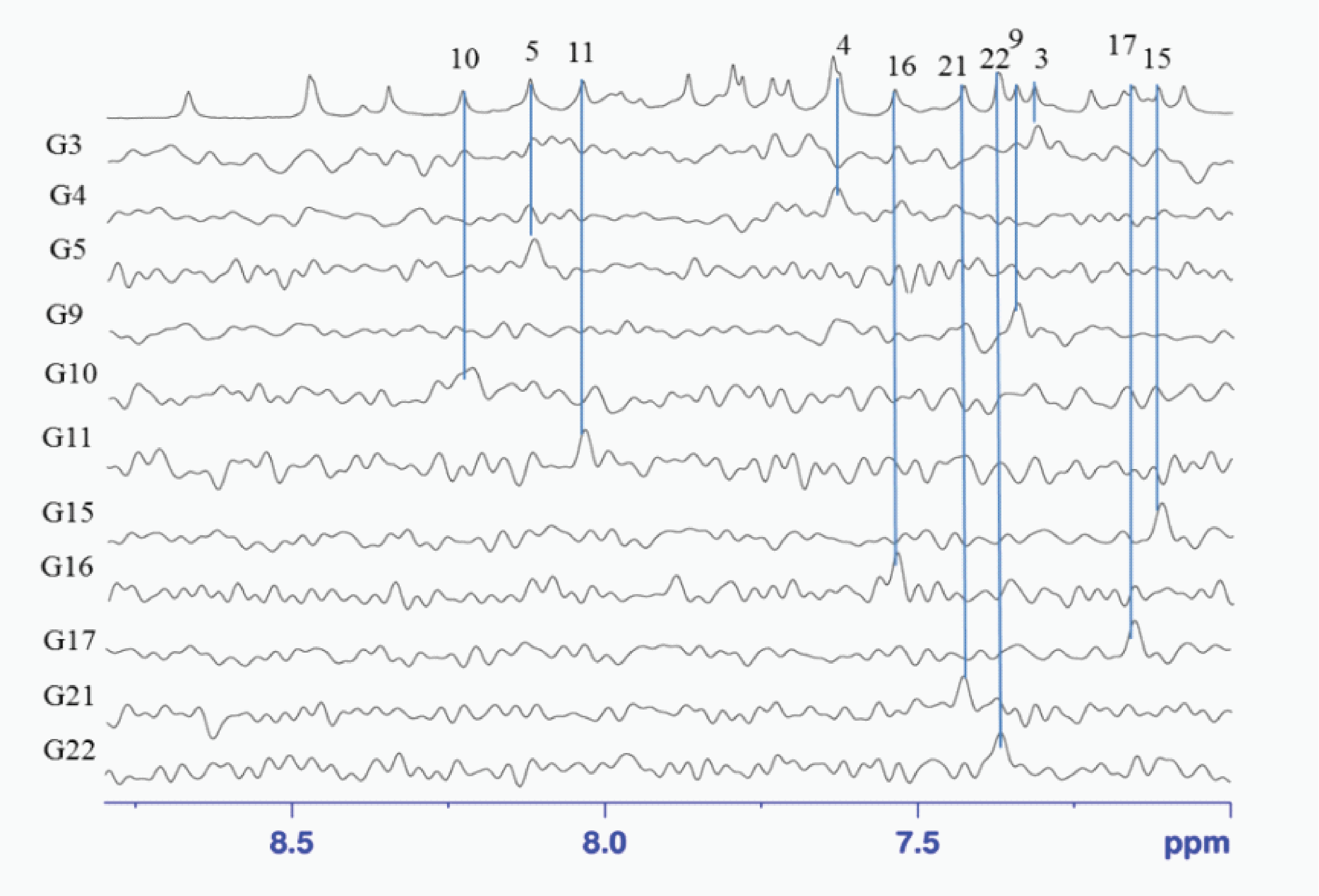

Figure S2. Long-range coupled 1D ^{15}N-^{1}H SOFAST-HMQC spectra of Tel23 G4. The H8 aromatic protons can be correlated and assigned by setting the magnetization transfer delay to 66.7 ms ($^{2}J_{NH}$ = 15Hz). Each sample contains 100 μM with 6% ^{15}N-labeled guanine at the indicated positions.

Figure S3

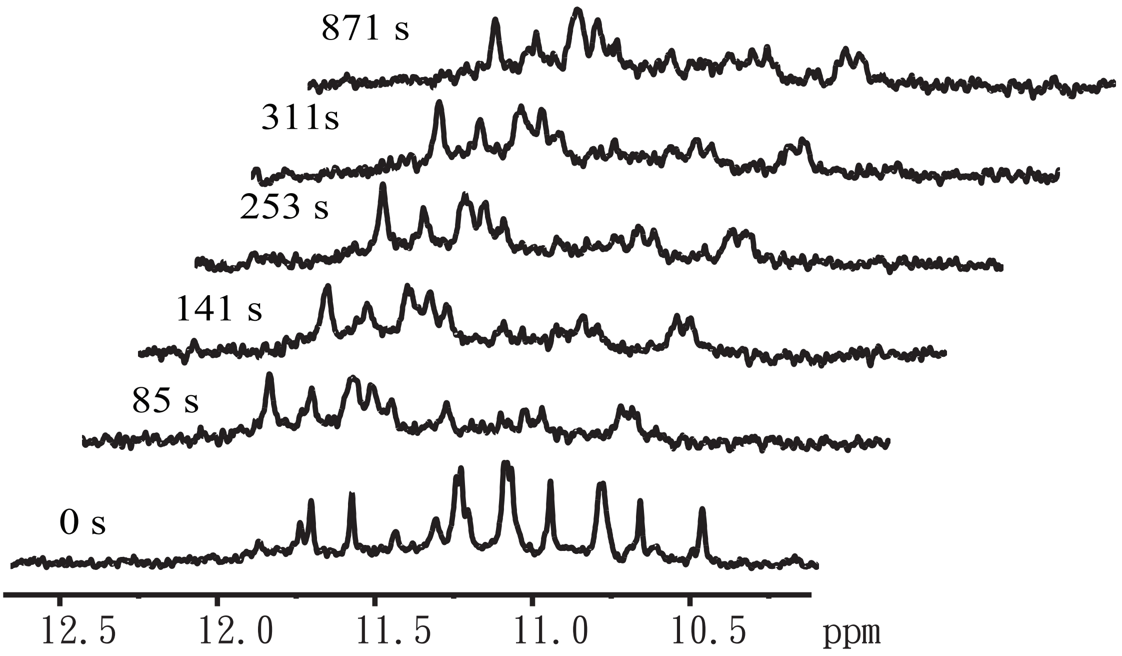

Figure S3. Time-dependant experiment of NMR at 25℃. The time-dependent imino proton spectra were acquired at given time point after adding K^+ within 60 s dead time and with 25 s in between each spectrum.

Figure S4

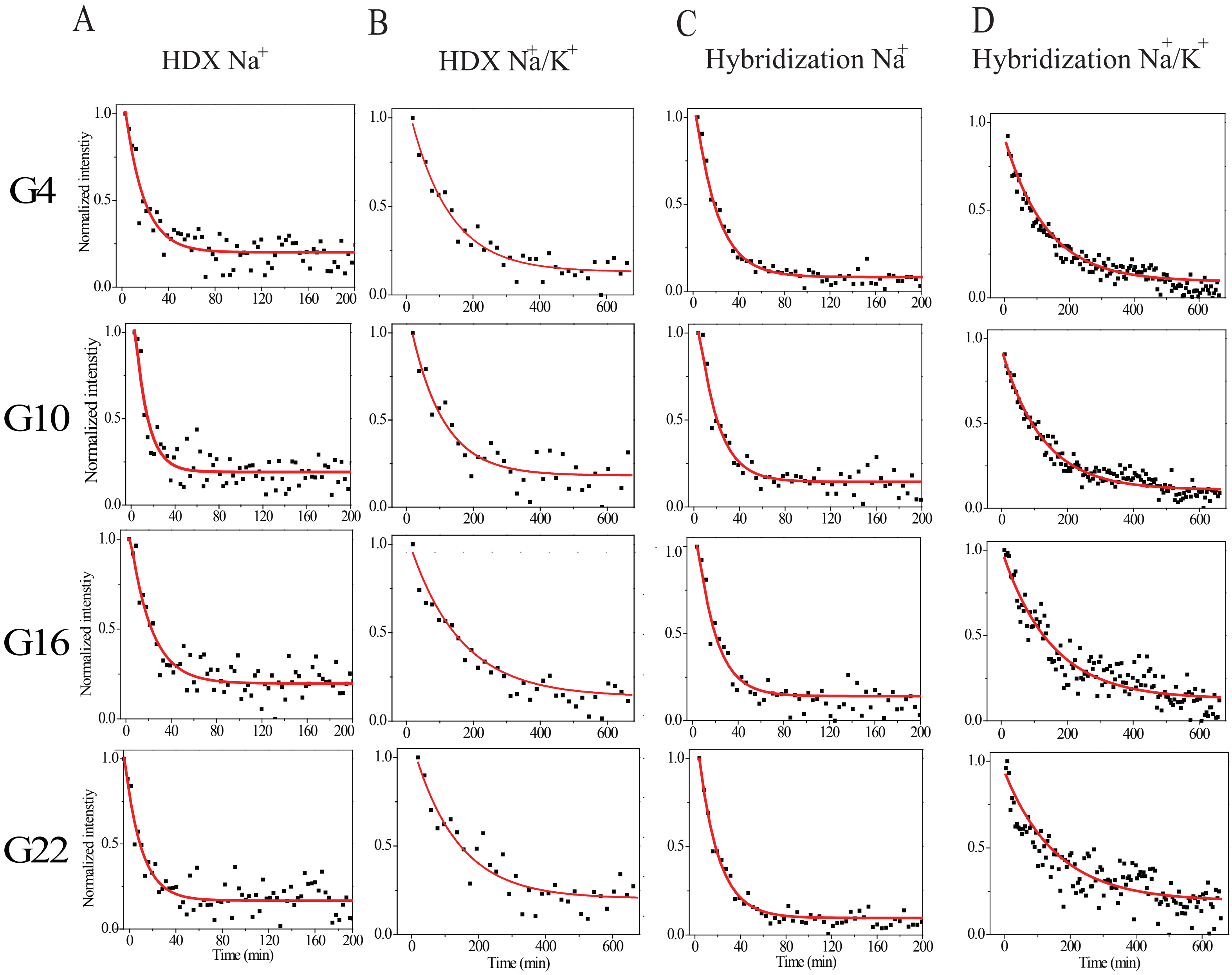

Figure S4. The decrease of imino proton resonance (black dot) of central G-tetrads (G4, G10, G16, and G122) fitted to a single exponential decay time (red line). HDX kinetics of Tel23 G4 Na^+ form (A) and a simultaneous Na^+/K^+ form (B). The hybridization kinetics of Tel23 G4 Na^+ form (C) and a simultaneous Na^+/K^+ form (D).

Figure S5

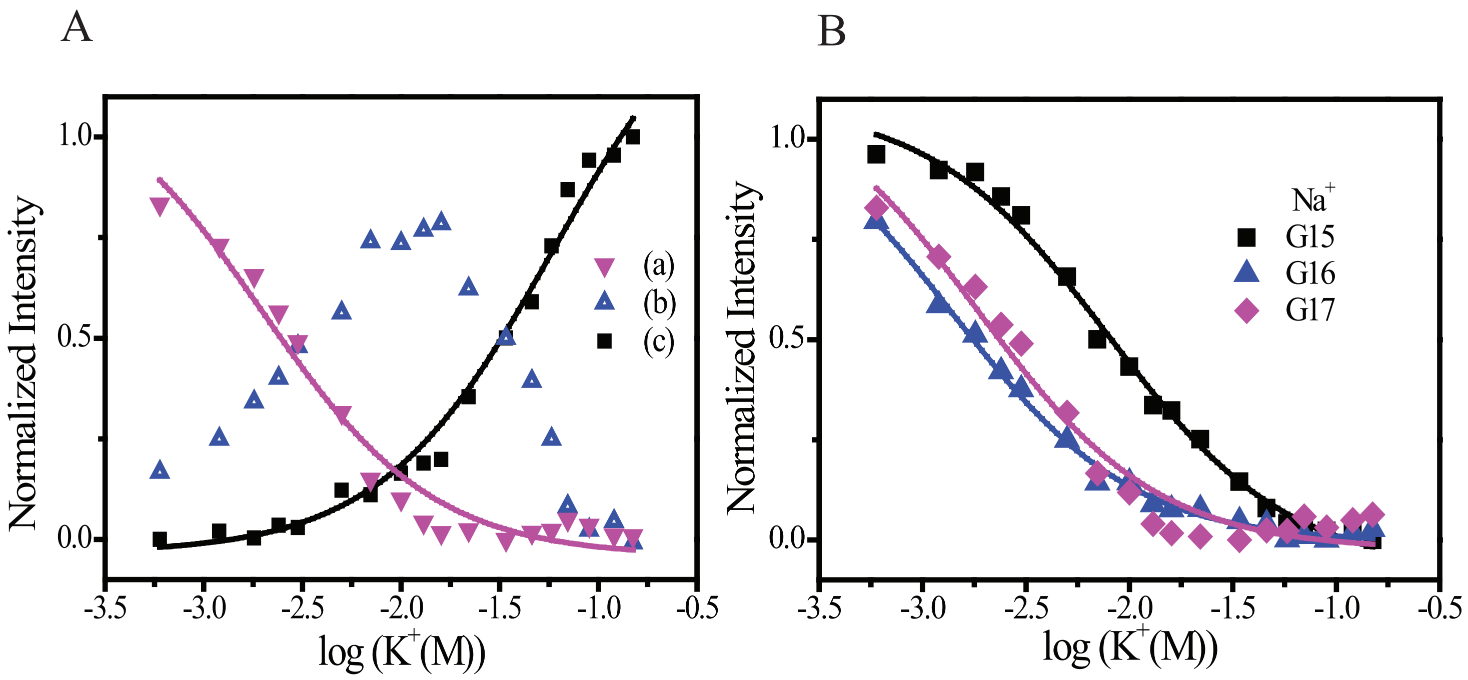

Figure S5. The imino protons normalized intensity plotted against the log-function of different K^+ concentrations. (A) The imino proton intensity of G4 in Na^+ as (a) (magenta), K^+ form as (c) (black), and the subtraction of (a) and (c) from total folded G4 structure will give (b) as the intermediate (blue). (B) The imino proton intensity of G15 (black), G16 (blue), and G17 (magenta) in Na^+ form.

Table S1

		Na^+ form relative distance (Å)	K^+ form relative distance (Å)	Na^+/K^+ form relative distance (Å)
(a)	G17H8-G21H1	4.9±0.1	3.9±0.4	3.9±0.4
	G3H8-G9H1	5.0±0.1	4.8±0.4	4.6±0.4
	G21H8-G3H1	5.1±0.1	4.7±0.3	4.5±0.3
	G9H8-G17H1	5.2±0.3	4.8±0.3	4.4±0.3
(b)	G22H8-G16H1	5.1±0.1	4.7±0.3	4.1±0.3
	G10H8-G4H1	5.0±0.2	4.6±0.3	4.2±0.3
	G4H8-G22H1	5.3±0.1	4.9±0.4	4.6±0.4
	G16H8-G10H1	5.2±0.2	5.1±0.5	4.7±0.5
(c)	G5H8-G23H1	5.5±0.2	4.4±0.2	4.2±0.2
	G11H8-G5H1	4.9±0.2	4.2±0.3	3.6±0.3
	G15H8-G11H1	5.1±0.3	4.8±0.4	4.5±0.4
	G23H8-G15H1	5.1±0.2	4.0±0.4	4.7±0.2
	Sum	5.1±0.2*	4.3±0.4*	4.3±0.4*

		Na^+ form relative distance (Å)	K^+ form relative distance (Å)	Na^+/K^+ form relative distance (Å)
(a)-(b)	G3H1-G22H1	n.d.	4.0±0.3	3.7±0.4
	G4H1-G9H1	4.2±0.4	4.0±0.3	3.6±0.4
	G21H1-G16H1	4.3±0.2	4.3±0.2	4.2±0.4
	G17H1-G10H1	4.4±0.2	n.d.	n.d.
(b)-(c)	G15H1-G16H1	5.1±0.4	4.5±0.2	3.8±0.4
	G22H1-G15H1	5.1±0.4	4.5±0.2	4.4±0.3
	G22H1-G23H1	5.5±0.3	4.6±0.2	4.4±0.4
	G4H1-G5H1	n.d.	4.3±0.2	4.0±0.2
	G4H1-G23H1	5.6±0.3	n.d.	n.d.
	Sum	4.9±0.6*	4.3±0.4*	4.0±0.3*

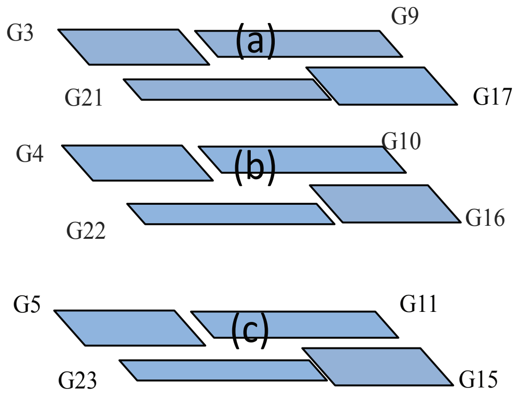

Table S1. The list of inter-proton distances between guanine imino protons, guanine imino to H8 protons on same G-tetrads (a, b, c), and adjacent G-tetrads (a to b, b to c) from NOESY of Tel23 in Na^+, K^+, and Na^+/K^+ forms. The base distance error of each G base was calculated from noise contribution of NOE signal whereas the asterisk on the average distance was the standard deviation of all distance in the column.

Table S2

	Glycosidic conformation	Na^+ form relative distance (Å)	K^+ form relative distance (Å)	Na^+/K^+ form relative distance (Å)		Na^+ form relative distance (Å)	K^+ form relative distance (Å)	Na^+/K^+ form relative distance (Å)
G3H1'-H8	syn	2.9±0.1	3.1±0.1	2.9±0.1	A14H1'-G15H1	3.6±0.3	3.9±0.2	3.9±0.2
G4H1'-H8	anti	3.7±0.2	4.3±0.3	4.5±0.3	A2H8-G9H1	6.0±1.0	4.6±0.4	3.9±0.5
G5H1'-H8	anti	3.8±0.2	3.5±0.3	3.6±0.3	A20H8-G21H1	5.7±0.4	4.8±0.3	4.7±0.3
G9H1'-H8	syn	3.0±0.1	2.9±0.1	2.9±0.1	A2H8-G21H1	5.2±0.4	4.9±0.2	4.8±0.2
G10H1'-H8	anti	4.5±0.2	4.4±0.2	4.6±0.2	T1H1'-G9H1	3.6±0.3	4.2±0.3	3.8±0.3
G11H1'-H8	anti	4.1±0.2	4.2±0.2	4.5±0.2	T1H6-G9H1	5.1±0.4	4.8±0.4	4.6±0.4
G15H1'-H8	syn	3.0±0.1	3.0±0.1	3.1±0.1				
G16H1'-H8	syn	2.9±0.1	2.9±0.1	2.7±0.1				
G17H1'-H8	anti	3.9±0.4	3.3±0.3	3.2±0.3				
G21H1'-H8	syn	2.9±0.1	3.0±0.1	3.1±0.1				
G22H1'-H8	anti	4.1±0.3	3.6±0.3	4.0±0.3				
G23H1'-H8	anti	4.0±0.2	4.1±0.2	4.4±0.2				

Table S2. The list of inter-proton distances between guanine H8 to H1' protons, guanine protons to loop base on same G-tetrads (a, b, c), and adjacent G-tetrads (a to b, b to c) from NOESY of Tel23 in Na^+, K^+, and Na^+/K^+ forms.

Table S3

	K_1		K_2
G10(Na^+)	97.0(0.98)	G4(K^+)	2.6(0.98)
G9(Na^+)	80.1(0.98)	G9,10(K^+)	2.3(0.98)
G16(Na^+)	82.5(0.98)	G22(K^+)	2.7(0.99)
G17(Na^+)	58.5(0.97)	G3(K^+)	2.6(0.98)
G22(Na^+)	59.7(0.97)		
G4(Na^+)	54.3(0.99)		
G23(Na^+)	38.6(0.99)		
G11(Na^+)	88.8(0.98)		
G5(Na^+)	17.1(0.98)		
G15(Na^+)	14.4(0.99)		

Table S3. Equilibrium constants (K_1 and K_2) of the Na^+/K^+ exchange of individual imino protons. The data are fitted according to the function of one-site bind model, and fitting R^2 values are shown in parentheses.
